# Using a Videogame Intervention to Reduce Anxiety and Externalizing Problems among Youths in Residential Care: an Initial Randomized Controlled Trial

**DOI:** 10.1007/s10862-017-9638-2

**Published:** 2017-11-29

**Authors:** Angela A. T. Schuurmans, Karin S. Nijhof, Rutger C. M. E. Engels, Isabela Granic

**Affiliations:** 1 Research & Development, Pluryn, P. O. Box 53, 6500 AB Nijmegen, The Netherlands; 2 Behavioural Science Institute, Radboud University Nijmegen, P. O. Box 9104, 6500 HE Nijmegen, The Netherlands; 3 Trimbos Institute (Netherlands Institute of Mental Health and Addiction), P. O. Box 725, 3500 AS Utrecht, The Netherlands; 4 Present Address: Utrecht University, P. O. Box 80125, 3508 TC Utrecht, The Netherlands

**Keywords:** Randomized controlled trial, Residential care, Anxiety, Externalizing problems, Videogame intervention, Intellectual disability

## Abstract

Residential care is among the most intensive forms of treatment in youth care. It serves youths with severe behavioral problems and is primarily focused on targeting externalizing problems. Despite best efforts, effect sizes remain moderate, which may be due to the disregarding of internalizing symptoms – in particular anxiety - and to limitations regarding the delivery model of interventions. This initial randomized controlled trial (*n* = 37) aimed to examine the effectiveness of a biofeedback videogame intervention (*Dojo*) as an addition to treatment as usual for youths with and without intellectual disability (ID) in residential care with clinical levels of anxiety and externalizing problems. *Dojo* targets both anxiety and externalizing problems, and incorporates the principles of conventional treatment, while addressing its limitations. Youths were randomly assigned to play *Dojo* (eight 30-min gameplay sessions) or to treatment as usual (TAU). Measurements of anxiety and externalizing problems were conducted at baseline, posttreatment, and 4-months follow-up through youths’ self-report and mentor-report. Completers-only analyses revealed decreases in self-reported anxiety and externalizing problems, and mentor-reported anxiety at posttreatment for participants in the *Dojo* condition compared to the control condition. Only mentor-reported anxiety was maintained at follow-up. No effect was found for mentor-reported externalizing problems. These findings provided preliminary evidence that *Dojo* is a promising, innovative intervention that engages high-risk youths. Practical implications are discussed.

## Introduction

The most intensive form of interventions for youth is residential care, which is often seen as a last resort solution for youths who have not responded well to previous treatment programs. Residential care is an out-of-home placement that typically provides 24-h care and offers mental health services with the goal of preparing youths to re-enter society (Whittaker et al. 2015). Such an invasive intervention is restricted to serve only those most in need, which includes youths with severe, complex behavioral and emotional problems. Their problem behavior is often combined with psychiatric disorders and/or intellectual disabilities (ID; Frensch and Cameron 2002), and up to 90% has been exposed to traumatic experiences such as neglect and/or abuse (Briggs et al. 2012). Thus, youths in residential care often show many risk factors and few protective factors (e.g., supportive caregivers, structured home settings) for behavioral and emotional problems, which contributes to the development and maintenance of these problems (Pollard and Hawkins 1999; Steinberg and Avenevoli 2000).

Residential care has a twofold purpose: first, to provide a safe and structured living environment for the youths in their group homes within the institution, and second, to offer intensive treatment to target problem behavior. At the group homes, group home workers are substitute caregivers who model appropriate behavior, provide support, and encourage youths to use the strategies they have learned in therapy. Treatment usually consists of evidence-based interventions to reduce problem behavior and teach youths adaptive alternative behaviors (McCurdy and McIntyre 2004; Kok et al. 1991). However, despite all efforts, residential treatment remains only moderately effective and approximately 25% of the youths leave care prematurely (Harder et al. 2006). A meta-analysis on the effectiveness of residential treatment showed an average effect size of *d* = .36 in the reduction of behavior problems for residential care with evidence-based treatment compared to standard residential care (group home care without specific treatment; de Swart et al. 2012). That meta-analysis highlights the importance of providing youths with treatment during their stay in the institution, but despite these promising outcomes, the average effect size of evidence-based treatment in residential care remains modest (Cohen 1988).

There are in particular two concomitant factors that may impede treatment effectiveness in residential care. The first one is that residential treatment focuses mainly on externalizing problems and tends to overlook internalizing symptoms, while most youths also exhibit co-occurring internalizing symptoms (Granic 2014). Comorbidity rates are in particular high for anxiety – approximately half of all youths in residential care shows clinically elevated levels of anxiety (Connor et al. 2004). These anxious feelings direct youths’ focus towards potential threatening stimuli, which may result in direct, impulsive aggression as a defense to these – real or perceived – threats (Vitaro et al. 2006). Even when youths do not show direct aggression, they tend to amplify their anxious feelings and maintain their state of arousal, rather than applying successful emotion-regulation strategies. This sustained attention to potential threats exhausts youths, and may eventually lead to externalizing behavior through an indirect route – the loss of inhibitory control (Granic 2014). Notably, for both the direct and the indirect route to aggression, anxiety is hypothesized as the eliciting mechanism that leads to youths’ inability to regulate their emotions. Thus, by ignoring anxiety in residential treatment, we may treat the symptoms while avoiding the causes of the behavior problems.

Another factor that may account for the modest effect sizes is that residential treatment usually consists of interventions based on cognitive-behavioral therapy (CBT) principles (de Lange et al. 2013). Although CBT is among the most effective forms of treatment in residential care (Garrido and Morales 2007; de Swart et al. 2012), it has some overarching limitations. These are limitations regarding the delivery model of CBT, not the principles themselves (Kazdin and Blase 2011; Kazdin and Rabbitt 2013). Not only should residential treatment focus on both internalizing and externalizing problems to improve its effectiveness, it should also be delivered in a way that is targeted towards youths’ needs.

Youths in residential care are often characterized by a lack of motivation to change their behavior (van Binsbergen 2003). Poor motivation is the key predictor for both low treatment effectiveness (Harder et al. 2012) and treatment dropout (Harder et al. 2011). In order to ensure that these youths do not leave care prematurely or drop out of intervention programs, these programs need to be engaging for youths. A new approach and potential solution for the delivery of youth interventions is the use of videogames. Whereas CBT depends largely upon imparting psychoeducational information, a didactic style of learning that contains few elements that are intrinsically motivating, videogames are able to deliver evidence-based techniques in an appealing context and make use of youths’ intrinsic motivation to engage them into treatment.

Also, psychoeducational CBT offers knowledge with limited opportunities to practice. Due to this gap between knowledge and behavior, the generalizability of CBT is limited. Youths usually know about appropriate, prosocial behavior, but in their everyday lives they often act impulsively and based on their emotions. Although CBT often incorporates exercises such as role-playing (Kendall et al. 2003), this rarely manages to provoke genuine emotions. Videogames, however, provide youths with the opportunity to learn by doing instead of memorizing (Vygotsky 1978), and are better able to elicit authentically emotional experiences. Youths are provided with an in-game environment where acquired techniques and strategies can be practiced until they are automatized and ideally can be generalized outside the game (Granic et al. 2014). The repetitive nature of gameplay fosters long-term learning (e.g., Rosas et al. 2003). In particular games that implement biofeedback may promote self-regulation skills and foster generalization of learned behaviors to youths’ daily lives (Yucha and Mongomery 2008). A videogame named *RAGE-Control* has successfully been integrated into a traditional CBT-based intervention to improve emotion-regulation among youths in residential care. *RAGE-Control* was effectively used to practice and strengthen the techniques learned during therapy sessions with a therapist (Ducharme et al. 2012; Kahn et al. 2013).

Another factor that impedes the effectiveness of CBT is that 20–25% of all youths in residential care is diagnosed with ID (van Nieuwenhuijzen 2010). These youths have limited social, emotional, and cognitive capacities (Magiati et al. 2014), while learning about cognitive biases and the links between feelings, thoughts, and behavior requires high-level processing. Playing a videogame, on the other hand, usually requires less cognitive load. Youths with ID might benefit more from experience-based interventions such as videogames compared to verbally based interventions such as CBT (Association for the Treatment of Sexual Abusers 2015). Treatment should be tailored to the specific needs of these youths and requires more simplistic language, smaller learning steps, and more emphasis on generalization to their real lives than conventional programs offer (Didden 2006). At the time, there is little evidence that conventional CBT alone has any beneficial effects on youths with ID (Sturney and Hamelin 2014; Taylor 2002). Although traditional CBT may require some adaptations for youths with ID, its principles have successfully been adjusted for their treatment. CBT components were made more concrete by implementing exposure and using relaxation techniques (i.e., deep breathing, muscle relaxation) to promote self-regulation among youths with ID (Shenk and Brown 2007).

The present study tested the initial effectiveness of *Dojo*, a biofeedback videogame intervention (developed by GameDesk, Los Angeles, CA), which targets the emotion-regulation problems that are hypothesized to underlie both anxiety and externalizing problems. The game consists of three in-game rooms (fear, frustration, and anger), each with one or two tutorials and an emotion-evoking mini game. The tutorials teach CBT-based relaxation techniques as deep-breathing techniques, progressive muscle relaxation, positive thinking, and guided imagery (Albano and Kendall 2002; Glancy and Saini 2005; Rapee et al. 2000; Sukhodolsky and Scahill 2012; Weisz and Kazdin 2010). The mini games are designed to trigger the emotion in question and challenge the youths to practice the newly acquired relaxation strategies in the in-game environment. This way, youths are playfully trained how to cope with their emotions. While playing the games, the players heart rate is monitored through biofeedback hardware and displayed on the screen, thus providing the player with real-time feedback on their stress levels. Controlling physiological reactions is required for success in the game, which encourages players to effectively regulate their emotions by using players’ desire to perform well in the game. For a more detailed description of the game, see Schuurmans et al. (2015). A recent pilot study demonstrated the feasibility and potential of *Dojo* as an intervention for a high-risk adolescent target population (Schuurmans et al. 2015). User evaluations and self-reported compliance for the tutorials were high, and initial outcome results on reductions in anxiety and externalizing problems were promising.

The present study was designed as an initial randomized controlled trial (RCT) to test the effectiveness of Dojo as an intervention for youths with clinical anxiety and externalizing problems in residential care. We hypothesized that participants who played *Dojo* as an addition to their treatment as usual (TAU) would show reduced levels of anxiety and externalizing problems compared to participants who received TAU alone. First, we focused on the main outcomes of the trial, the immediate posttreatment effects on symptoms of anxiety and externalizing problems. Then, we examined intervention outcomes at 4-months follow-up.

## Materials and Method

### Design and Procedure

The present study utilized a RCT design with two parallel conditions (*Dojo* versus TAU) and was conducted in residential institutions that provide (secure) youth care for youths with and without ID. In these institutions, youths live in group homes consisting of six to ten youths, with group home workers as substitute care givers. Participants were recruited by clinicians. Inclusion criteria consisted of clinically elevated levels of both anxiety and externalizing problems, based on clinician assessment. Participants were excluded if they were diagnosed with an Autism Spectrum Disorder or exhibited psychotic symptoms, and in one case, based on the clinician advising against study participation. This participant showed severe anxiety specifically for ghosts, which made the clinician fear that participation would have a negative effect. Next, participants were invited for an individual meeting during which they were informed about the study and were asked for their written consent. It was explained that they could quit the study at any time and that all information would be treated confidentially. For participants younger than the age of 16, their legal guardians were also informed and asked for written consent. All participants received ten euros for their participation. Ethical review and approval were provided by the Faculty of Social Sciences, Radboud University Nijmegen (ECSW2013–1811-154) and the procedure was registered in the Trial Register for RCTs (www.trialregister.nl; Trial ID: NTR4477).

Assessments were conducted for both conditions at three time points: week 1 – prior to the intervention (i.e., baseline), week 5 – immediately following the intervention (i.e., posttreatment), and at 4-months follow-up. Measurements consisted of participants’ self-report and mentor-report (the group home worker with whom they had the most contact). The self-report measures were completed in an interview format to ensure comprehension (in particular participants with ID had difficulties with reading). The interviews took 15 to 20 min and were conducted by the first author or a research assistant.

### Intervention

#### Experimental Condition (*Dojo*)

Participants in the experimental condition received the *Dojo* intervention as an addition to their usual treatment program. The intervention consisted of eight 30-min sessions during which participants played *Dojo* on a laptop. The sessions took place twice a week for four consecutive weeks in an office at the group homes or in a therapist office located on the campus of the residential institution. The game sessions were led by the first author and two research assistants who were trained to explain the game to participants and guide them through the tutorials and challenges according to a standardized protocol. Critically, in each session, participants were instructed to complete the tutorial – thus, to practice the relaxation technique – before they were allowed to start with the matching mini game.

#### Control Condition (TAU)

The TAU condition was designed to reflect standard practice. Participants in both conditions received TAU; treatment as recommended by their clinicians regardless of this study. There were no restrictions for the type of interventions participants received, we only kept track of it. Individual therapy (e.g., CBT) and/or medication (e.g., Ritalin) were the most received interventions. Some participants received group therapy (e.g., social skills training) and/or family therapy (e.g., multisystematic therapy; see Table 1).

**Table 1:** Participants’ characteristics (*N* = 37)

	*Dojo* condition (*n* = 18)	Control condition (*n* = 19)
Mean age (*SD*)	13.67 (1.82)	14.26 (1.94)
Gender – *n* (%)		
Male	14 (77.8%)	17 (89.5%)
Female	4 (22.2%)	2 (10.5%)
Comorbid diagnosis – *n* (%)		
ADHD	7 (38.9%)	5 (26.3%)
Trauma	2 (11.1%)	6 (31.6%)
Developmental disorder	1 (5.6%)	1 (5.3%)
Intellectual disability – *n* (%)		
None	9 (50%)	10 (52.6%)
Mild	3 (16.7%)	3 (15.8%)
Moderate	6 (33.3%)	5 (26.3%)
Severe	–	1 (5.3%)
Received other interventions during study – *n* (%)		
Individual therapy	9 (50%)	9 (47.4%)
Group therapy	4 (22.2%)	1 (5.3%)
Family therapy	3 (16.7%)	5 (26.3%)
Medication	8 (44.4%)	8 (42.1%)
Weekly hours videogame play* – *mean* (*SD*)	12.61 (16.29)	11.88 (11.03)

*SD*, standard deviation; ADHD, attention deficit hyperactivity disorder

*Indicating previous general gaming experience before the start of the study

### Randomization

Randomization of the participants to one of the two conditions (*Dojo* versus TAU) was stratified by gender and intellectual disability level (none/mild/moderate) to ensure equal ratios of participants in both conditions. The first author randomly assigned participants to the conditions using a computer-generated list of random numbers. Randomization was executed before we contacted the youths to ask whether they were willing to participate in the study. Participants were not informed about their condition before they decided whether they wanted to take part in the study. Then, participants in the *Dojo* condition were told that *Dojo* was designed to help them regulate their emotions. Participants in the control condition were told that they could play *Dojo* for treatment purposes after the follow-up measurement.

### Sample Size

G*Power 3 (Faul et al. 2007) was used to estimate our targeted sample size, based on a small-to-medium effect of *d* = 0.36 (de Swart et al. 2012), an alpha of 0.05, a power of 0.80, and an estimated correlation of 0.7. A pre-post (within) by two groups (between) ANOVA would require a total sample of 40 participants.

### Participants

**Fig. 1:**
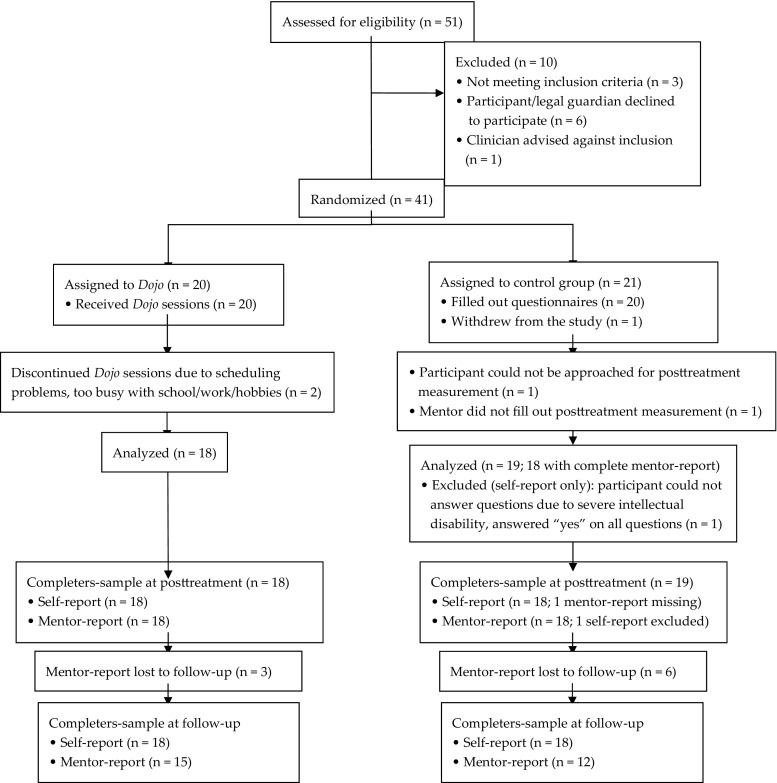
Flow diagram

Participants were recruited at two residential institutions from March 2014 to June 2014. We recruited 51 participants, of which ten were excluded before randomization because they did not meet the inclusion criteria or the participant/guardian declined to participate. At baseline, our sample consisted of 41 participants. We compared participants’ self-reported baseline scores on anxiety and externalizing problems with the self-reported scores of healthy Dutch adolescents within a similar age range (Muris et al. 2002; van Widenfelt et al. 2003). The mean total scores on anxiety were 19.33 for the complete sample, 18.13 for males, and 24.00 for females. In the sample of Muris et al. (2002), these scores were respectively 16.9, 12.7, and 20.4. It was noted that the males in our study report high anxiety scores in particular. The mean total scores on externalizing problems were 10.78 for the total sample, 10.90 for males, and 10.17 for females. Van Widenfelt et al. (2003) reported respectively 7.65, 8.0, and 7.3. Attrition was low with only four participants withdrawing from the study during the intervention (see Flow diagram, Fig. 1). Baseline demographic characteristics are shown in Table 1. No statistical differences were found between the experimental and control condition on any of these baseline factors, including sex, age, comorbid diagnoses, intellectual disability, previous gaming experience, and the type of interventions participants received during the study (all *p* > .10).

### Measurements

#### Anxiety

Self-reported and mentor-reported anxiety was measured using the total scores of the Dutch version of the Spence Children’s Anxiety Scale (SCAS; Spence 1998). The SCAS has 45 four-point items (e.g., I worry about things, I am scared of the dark) and is composed of five subscales: ‘separation anxiety’, ‘social phobia’, ‘obsessive–compulsive disorder’, ‘fears of physical injury’, and ‘generalized anxiety’. Cronbach’s alpha of the SCAS measurements were .88, .92, and .87 (self-report), and .88, .89, and .92 (mentor-report) for the baseline, posttreatment, and follow-up measurement, respectively. The SCAS has good reliability and validity and shows high correlations with other anxiety questionnaires (Muris et al. 2002). The SCAS has not yet been tested among youths with ID but is suitable for children as young as 8 years old (Spence 1998) so no problems were expected in a sample of adolescents with ID.

#### Externalizing Problems

Self-reported and mentor-reported externalizing problems were measured using the Dutch version of the Strengths and Difficulties Questionnaire (SDQ; Goodman 1997; van Widenfelt et al. 2003). We used the externalizing subscales ‘conduct problems’ (e.g., I fight a lot), ‘hyperactivity-inattention’ (e.g., I am easily distracted), and ‘peer problems’ (e.g., I am usually on my own), each consisting of five three-point items. We calculated a total score of externalizing problems by summing up these three subscales. Cronbach’s alpha of this externalizing problems score were .81, .83, and .68 (self-report), and .75, .69, and .66 (mentor-report) for the baseline, posttreatment, and follow-up measurement, respectively. The SDQ was successfully used in a sample of youths with ID (Kaptein et al. 2008), has good validity, and correlates highly with other established questionnaires that measure externalizing problems (Muris et al. 2003).

#### User Evaluation

To measure the appeal of the videogame intervention, we asked participants in the *Dojo* condition to rate statements regarding their satisfaction with the game on a 5-point scale (e.g., I liked playing *Dojo*). Participants’ compliance was assessed by self-report on a 7-point scale (e.g., How would you rate your compliance during the muscle relaxation training?). Finally, we asked participants which of the relaxation techniques they used most in their daily lives (participants were allowed to choose more than one answer).

#### Gaming Experience

At baseline, participants were asked for their previous gaming experience. A pilot study (Schuurmans et al. 2015) showed that it was difficult for participants with ID to come up with an estimation of the average hours per week they play videogames, so this measurement was divided in average amount of hours they played videogames: (1) on week days at the residential center, (2) during the weekend at the residential center, and (3) during the weekend when participants left the residential center and stayed at home. Based on these three estimations, we calculated the average hours of gameplay a week for each participant.

### Statistical Analyses

Our main outcomes were immediate intervention effects on (1) anxiety, and (2) externalizing problems. Additionally, we examined intervention outcomes at 4-months follow-up. We performed completers-only analyses, i.e. involving only those participants who completed the measurements. Outcome data were missing for 12.2% of the posttreatment measurements (both self-report and mentor-report), 12.2% of the self-reports at follow-up, and 34.1% of the mentor-reports at follow-up. Although missing data usually are imputed for analyses, imputation of missing data that are not missing at random may lead to misleading results and an even bigger bias than the analyses of complete cases only (Sterne et al. 2009). Our missing data were not missing at random, in particular the mentor-reports at follow-up: the missing cases were either participants who improved in their behavior and returned home, or participants whose behavior deteriorated and who were replaced to a secured institution.

We calculated descriptive statistics (means and standard deviations) for participants’ baseline characteristics, anxiety and externalizing problems separately for both conditions, and for user evaluations for participants in the *Dojo* condition. Also, correlations, independent *t*-tests and chi square tests to test for differences between conditions (*Dojo* versus TAU) at baseline were conducted. For the main effect analyses (anxiety and externalizing problems) we used a mixed-model repeated measures analysis of variance (ANOVA) with time as within-groups factor and condition as between-groups factor. Analyses were conducted comparing change from baseline to posttreatment, and from baseline to follow-up for the two conditions. The partial eta squared (η^2^_p_) was derived in order to estimate the magnitude of the difference between groups and the effect size of the intervention. Cohen’s *d* effect size values (see Cohen 1988) were calculated for the within-groups effect sizes for change from baseline to posttreatment and from baseline to follow-up.

## Results

### Main Outcomes at Posttreatment and Follow-Up

Baseline, posttreatment, and follow-up scores on self-reported and mentor-reported anxiety and externalizing problems are presented in Table 2. There was no significant difference on baseline scores (all *p* > .35) between the conditions. Table 2 also includes the statistics of the main outcome analyses. Significant interactions were reported for self-reported anxiety and externalizing problems at posttreatment, and mentor-reported anxiety at both posttreatment and follow-up. Effect sizes for these interactions ranged from small to medium (Cohen 1988). After the main analyses, we conducted additional analyses where we controlled for ID. There was no significant effect of ID on the intervention outcomes (all *p* < .10).

**Table 2:** Outcomes at baseline, posttreatment, and 4-months follow-up (*N* = 37)

Measurement	*Dojo* condition (*n* = 18)		Control condition (*n* = 19)*					
	*Mean* (*SD*)	*d*	*Mean* (*SD*)	*d*	*df*	*F*	*p*	η^2^_p_
Baseline								
Externalizing problems self-report	10.22 (6.00)		11.50 (5.89)					
Anxiety self-report	21.17 (14.55)		16.94 (14.83)					
Externalizing problems mentor-report	13.39 (5.37)		15.11 (4.23)					
Anxiety mentor-report	17.50 (10.60)		18.83 (7.94)					
Posttreatment								
Externalizing problems self-report	8.00 (5.08)	.36	12.28 (4.98)	.22	34	4.17	.049	.11
Anxiety self-report	16.44 (16.30)	.33	18.67 (16.50)	.13	34	6.28	.017	.16
Externalizing problems mentor-report	14.17 (5.07)	.20	14.56 (3.94)	.29	34	1.99	.168	.06
Anxiety mentor-report	13.61 (9.47)	.42	19.11 (7.85)	.14	34	5.28	.028	.14
Follow-up								
Externalizing problems self-report	8.17 (3.92)	.50	12.39 (3.33)	.25	34	3.91	.056	.10
Anxiety self-report	16.28 (15.29)	.35	17.89 (10.50)	.08	34	1.25	.272	.04
Externalizing problems mentor-report	14.17 (3.64)	.23	15.00 (4.85)	.00	19	.27	.610	.01
Anxiety mentor-report	13.92 (12.15)	.36	13.70 (5.72)	.38	19	2.53	.128	.12

*d* = within-groups effect size, Cohen’s d. * self-report *n* = 18, mentor-report *n* = 18. The main analyses were also conducted with the intention-to-treat sample were missing data were imputed (*N* = 41), which resulted in statistical differences for self-reported externalizing problems at posttreatment (*p* = .101) and follow-up (*p* = .031), self-reported anxiety at posttreatment (*p* = .031), and mentor-reported anxiety at follow-up (*p* = .005)

### User Evaluations

All participants in the *Dojo* condition attended the eight scheduled gameplay sessions, but for four participants it took 5 weeks to complete the sessions due to scheduling problems. Participants reported high satisfaction with *Dojo*: evaluation scores were 4.53 out of 5 for ‘liked playing *Dojo*’ (*SD* = .62), 4.00 for ‘thinks other youths will like playing *Dojo*’ (*SD* = .92), 3.88 for ‘liked *Dojo* being a videogame intervention’ (*SD* = 1.22), and 4.53 for ‘*Dojo* is useful in daily life (*SD* = 1.07). Participants also reported high compliance during the relaxation tutorials. The mean scores for self-reported effort is 5.76 out of 7 for positive self-talk, (*SD* = 1.15), 6.12 for muscle relaxation (*SD* = .99), 5.95 for guided imagery (*SD* = 2.00), and 6.06 for deep-breathing techniques (*SD* = 1.09). The relaxation techniques that were rated as most used in participants’ daily lives are deep-breathing relaxation (64.7%) and positive thinking (47.1%).

## Discussion

### Key Findings

The current study utilized an initial RCT to test the effectiveness of *Dojo* as an intervention for high-risk youths with clinical anxiety and externalizing problems in residential institutions. We expected that eight sessions of *Dojo* gameplay would lead to reduced levels of anxiety and externalizing problems; these hypotheses were partly supported. Youths who played *Dojo*, compared with youths in the control condition, showed reductions in self-reported anxiety and externalizing problems at posttreatment, and mentor-reported anxiety at both posttreatment and follow-up. Contrary to our expectations, results showed no differences between conditions in self-reported anxiety and externalizing problems at follow-up, and mentor-reported externalizing problems at both posttreatment and follow-up. These findings indicate that the intervention resulted in an immediate reduction of anxiety, and from youths’ own perspective, also externalizing problems.

We have to be cautious with our conclusions regarding these outcomes, given the small sample size and subsequent low power in this study may have resulted in less robust results. The mentor-reported follow-up results have to be interpreted cautiously due to the high attrition rates. This was due to participants leaving the institutions. Participants were either discharged from the institutions to return home, because they showed improvements in their behavior (*n* = 3), they were replaced to other, secured institutions, because their behavior problems deteriorated (*n* = 6), or they refused further treatment and left the institutions without being discharged (*n* = 1). Whereas all participants themselves were willing to complete the last interview for this study, even when they left our institutions, we were unable to obtain mentor-reports for these participants. This non-random missing mentor-report data at follow-up may have resulted in an unrepresentative sample (Graham 2009). Nevertheless, we decided to report these results, but we have to refrain from drawing firm conclusions from this measurement.

Although youths themselves report a decrease in externalizing behavior at posttreatment, their mentors do not report any effect on externalizing problems. These variations in outcomes might be caused by a difference in perception between youths and their mentors. Other studies that were conducted in residential institutions have reported comparable results, with substantial disagreement between youths’ self-reports and mentor-reports, in particular for outcomes on externalizing problems (Bastiaansen et al. 2004; Grills and Ollendick 2003; Nijhof et al. 2011). It may be that group home workers are more critical in the assessment of youths’ behavioral progress compared with youths themselves, precluding them from noticing changes (Knorth et al. 2008).

Our results suggest that *Dojo* has a larger effect on anxiety than on externalizing problems. When we compare *Dojo* with conventional interventions that target anxiety and externalizing problems, there is a larger overlap with traditional components of anxiety treatment (i.e., skills training such as affect recognition, relaxation, and exposure; e.g., Kendall et al. 2003) than with aggression treatment (i.e. teaching prosocial behavior, anger control, and moral reasoning; Goldstein and Glick 1987). Although *Dojo* has one room that specifically targets anger, the main idea of the game is to teach youths to recognize their emotional and physical arousal, and to control this by practicing relaxation techniques.

Surprisingly, youths’ immediate decrease in self-reported anxiety and externalizing problems was not maintained at follow-up, only mentor-report anxiety showed an effect at both posttreatment and follow-up. This suggests that although *Dojo* could be an effective way to decrease anxiety and externalizing problems, from the youths’ perspective, the intervention may not help them to cope with future anxiety-provoking situations. In order to maintain immediate posttreatment effects, it may be necessary to provide youths with a ‘booster session,’ as done by *The Growth Factory*, a computerized mindset intervention developed for youths in residential care (Helmond et al. 2014).

Finally, we would like to discuss our results in the light of the findings of Scholten et al. (2016), who also tested *Dojo*, but as a method of prevention in a non-clinical sample of adolescents at risk for anxiety. They compared *Dojo* to the commercial videogame *Rayman*, and found equal reductions in anxiety for both conditions. This could mean that both videogames were equally effective in reducing anxiety, or that neither was effective. This question remains unanswered due to the lack of an inactive control group, but even when we assume that *Dojo* did not have an effect in their study, there are some explanations for the different results in our study. It could be due to the substantial differences between the two target populations. It may be that *Dojo* does work as an intervention for a clinical population with and without ID, but not as prevention for non-clinical adolescents. Another possibility is the difference in the ways *Dojo* was delivered. Scholten et al. (2016) allowed youths to play freely with minimal supervision, which made it possible for youths to skip the relaxation tutorials – which are hypothesized as *Dojo’s* working mechanisms – and play the mini games only.

### Strengths and Limitations

To our knowledge the current study is the first to examine a videogame intervention in a residential treatment setting with youths with and without ID. Research in this clinical, high-risk context is critical to establish intervention effectiveness and requires minimal translation to implement its results in these settings. Attrition in high-risk samples is usually high, but we lost only two participants in each condition from baseline to posttreatment. Both participants in the *Dojo* condition decided to quit the sessions due to scheduling problems: the sessions had to be scheduled after school hours and interfered with their part time job and/or leisure activity appointments. One participant asked for the opportunity to quit the study but start again with the *Dojo* sessions after the summer, since he would have more spare time by then. This indicates that although he quit the study, it was not because he was not motivated to play *Dojo*. Youths’ positive game evaluations demonstrated that they liked the game and they reported high compliance during the relaxation tutorials. Moreover, not only was treatment fidelity high – all participants completed the eight scheduled sessions – all youths in the control condition still wanted to play *Dojo* after the follow-up measurement. They did not receive an incentive for this, which indicated that they were intrinsically motivated to play. This suggests that we met our goal as for engaging this hard-to-motivate population into treatment.

The biggest limitation of the present study is its small sample size. Its results should be interpreted with caution, since these were not robust. Outcomes changed depending on whether or not the missing data were imputed. This study was not powered to definitively test the effectiveness of *Dojo*. In the future, rigorously designed and adequately powered RCTs could establish the effectiveness of *Dojo* on anxiety and externalizing problems and examine potential mediating mechanisms.

Participants in the *Dojo* condition received the intervention as an addition to TAU, while the control condition only received TAU. This means that participants in the *Dojo* condition received extra individual attention compared to the controls. Although active control groups are more rigorous, these are only superior when participants in both conditions have the same type of attention and the same expectations of improvement (Boot et al. 2013). Thus, optimal control would be a videogame that is comparable, but does not include the working mechanisms. This was impossible to achieve for us, since the study was conducted within institutions which had restrictions for casual videogame play. Clinicians did not agree on implementing a condition in which youths were allowed to play a videogame that was not expected to lead to mental health benefits. Moreover, the primary purpose of this study was to test the effectiveness of *Dojo* as a beneficial addition to regular treatment, not to determine its superiority to another form of treatment, which makes TAU a valid control condition (Freedland et al. 2011).

Our study design included not only a posttreatment measurement to assess immediate intervention effects, but also a 4-months follow-up measurement to evaluate whether effects were maintained over time. While attrition rates in these high-risk populations are in particular high for follow-up measurements, all participants who were included at posttreatment, also completed the self-report measurement at follow-up. However, we lost a substantial amount of the mentor-reports at follow-up, for reasons explained above.

The gameplay sessions were supervised by the first author and two research assistants, while an ideal clinical study design does not include the researcher’s involvement in the intervention sessions. Given limited funds and personnel time, it was not feasible to hire an additional research assistant blind to the study goals. Gameplay supervision was done following a standardized protocol, to prevent possible supervisor effects. Moreover, since *Dojo* teaches the emotion-regulation techniques within the game, the only task for supervisors was to answer any questions and to ensure that the youths followed the instructions (e.g., completing the tutorials before starting with the game).

Although this study showed the potential of *Dojo* as a form of treatment, we do not propose *Dojo* as a stand-alone intervention that is able to replace interventions already in use. *Dojo* has advantages compared to conventional treatment, but also possible disadvantages. For example, an important element of traditional therapy that may have positive effects is therapeutic alliance (Shirk and Karver 2003), something that is missing for *Dojo*.

## Conclusion

The findings of this study suggest that *Dojo* is worth further evaluation as an intervention for short-term reductions in anxiety and externalizing problems among high-risk youths in residential care. Playing *Dojo* resulted in reduced self-reported anxiety and externalizing problems, and mentor-reported anxiety at posttreatment. Of these three effects, only mentor-reported anxiety was maintained at follow-up. There was no effect on mentor-reported externalizing behavior. Although our results should be interpreted cautiously given the small sample size of this study, these findings contribute towards the evidence for a gaming approach in youth interventions. In the future, a RCT with a larger sample could be a useful next step. Not only would this allow mediator/moderator analyses, but *Dojo* could also be compared with active control groups, such as a closely matched control game or a standardized CBT-based intervention to assess whether *Dojo* is equally effective or perhaps even superior. To conclude, the results of this initial RCT suggest that *Dojo* may be an innovative, promising form of treatment for some of the most vulnerable and hard-to-treat populations in our society. A fully powered trial would be necessary to establish its effectiveness.
